# Global trends, risk factors, and therapeutic associations of fungal pulmonary infections in lung cancer: A systematic review and meta-analysis

**DOI:** 10.36416/1806-3756/e20250076

**Published:** 2025-11-19

**Authors:** Milad Sheervalilou, Mostafa Ghanei, Masoud Arabfard

**Affiliations:** 1. Chemical Injuries Research Center, Systems Biology and Poisonings Institute, Baqiyatallah University of Medical Sciences, Tehran, Iran.

**Keywords:** lung cancer, fungal infection, pulmonary infection, aspergillosis, Pneumocystis jirovecii, pneumonia

## Abstract

**Objective:**

Fungal pulmonary infections are a significant complication in lung cancer, adversely affecting prognosis and treatment outcomes. This meta-analysis aimed to estimate the prevalence of chronic pulmonary aspergillosis (CPA) and Pneumocystis jirovecii pneumonia (PJP) in lung cancer patients and to identify associated clinical predictors.

**Methods:**

A systematic search of EBSCOhost, Embase, PubMed/MEDLINE, Scopus, and Web of Science retrieved 2,823 records, of which 7 studies were eligible (PROSPERO: CRD42024551104). Meta-analyses of proportions and dichotomous and continuous variables were performed using R (meta package) via Jamovi and RevMan 5, with statistical significance set at p<0.05.

**Results:**

Among 15,901 lung cancer patients, 177 had CPA and 135 had PJP. The pooled prevalence was 1% for CPA and 23% for PJP. CPA was significantly associated with male sex, smoking, COPD, interstitial lung disease, tuberculosis, and squamous cell carcinoma, and negatively associated with adenocarcinoma. CPA patients also had significantly lower BMI. Bilobectomy, radiotherapy, and concurrent chemoradiotherapy were additional risk factors for CPA. High-dose corticosteroid use (≥20 mg/day) was significantly associated with PJP.

**Conclusion:**

CPA occurs in a clinically distinct subset of lung cancer patients with identifiable risk factors, while PJP appears to be strongly linked to immunosuppressive therapy. Improved screening strategies are warranted to mitigate the burden of these infections in vulnerable lung cancer populations.

## INTRODUCTION

Lung cancer remains the leading cause of cancer-related mortality worldwide. It originates from epithelial cells of the respiratory tract and is broadly classified as small cell lung cancer (SCLC) or non-small cell lung cancer (NSCLC), the latter encompassing subtypes such as lung adenocarcinoma (LUAD) and squamous cell carcinoma (SCC).^1^ Between 2001 and 2019, over 4 million U.S. patients were diagnosed,^2^ and 2.2 million new cases were reported globally in 2020.^3^ A 2023 meta-analysis estimated overall lung cancer mortality at 6-16%,^4^ though survival plummets to 18.6% in cases of metastatic disease.^5^

Fungal pulmonary infections (FPIs) are underrecognized yet critical complications in lung cancer, with profound effects on morbidity, treatment outcomes, and survival. Cancer-related immunosuppression-caused by chemotherapy, radiation, and targeted therapies-predisposes patients to opportunistic fungi such as *Aspergillus* spp., *Pneumocystis jirovecii*, and *Cryptococcus* spp. These pathogens exploit therapy-induced immune dysfunction, structural lung damage, and impaired mucociliary clearance to establish invasive or chronic infections.^6^ ^-^ ^11^ FPIs often mimic or aggravate cancer-related symptoms, leading to diagnostic delays and complex clinical management.^6^ ^,^ ^7^ ^,^ ^9^ ^-^ ^11^

Chronic pulmonary aspergillosis (CPA), a progressive infection caused by *Aspergillus* species, is particularly prevalent in this population. A Japanese multicenter study reported CPA-complicated lung cancer in patients undergoing anticancer treatment, correlating with poor prognostic factors such as squamous cell histology and low body mass index (BMI).^6^ While CPA itself was not a direct cause of mortality, it led to treatment interruptions (e.g., pneumonitis) and a median overall survival of 14.57 months.^6^ Similarly, a cross-sectional cohort detected *Aspergillus* colonization in 47.8% of newly diagnosed, non-neutropenic lung cancer patients, with *A. niger* as the dominant species. Notably, *A. niger* showed concentration-dependent cytotoxicity in human lung fibroblasts, suggesting a potential role in accelerating tissue damage and cancer progression.^7^ Post-surgical NSCLC patients receiving trimodality therapy also exhibited elevated CPA risk, particularly those with prior adjuvant chemotherapy or radiation pneumonitis.^10^ Localized CPA responded well to surgical or antifungal intervention, whereas disseminated disease was frequently fatal, underscoring the importance of early diagnosis.^10^

*Pneumocystis jirovecii* pneumonia (PJP) further complicates lung cancer management. A nested PCR study in Turkey detected *P. jirovecii* DNA in 66.7% of lung cancer patients-threefold higher than in non-cancer controls-with symptoms such as anorexia and weight loss strongly associated with colonization.^8^ These findings highlight the importance of systematic screening in symptomatic patients. Moreover, analysis of exhaled breath condensate identified *A. niger*, *A. ochraceus*, or *Penicillium* spp. in 27.9% of lung cancer patients, but not in healthy controls,^11^ suggesting environmental or host-related factors may predispose to FPIs. 

Despite these insights, significant knowledge gaps remain. Most available evidence stems from small, retrospective studies,^6^ ^,^ ^9^ ^,^ ^10^ limiting generalizability. FPIs-including CPA and PJP-are associated with prolonged hospitalization, treatment disruptions, and increased mortality, underscoring the need for greater clinical vigilance, routine screening in high-risk subgroups, and integrated management strategies.^6^ ^,^ ^7^ ^,^ ^10^ The present systematic review synthesizes current evidence on the prevalence, clinical predictors, and treatment-associated risk factors of FPIs in lung cancer patients, aiming to inform optimized diagnostic and therapeutic approaches.

## METHODS

### Review Question

The objective of this systematic review and meta-analysis was to determine the prevalence of FPIs in lung cancer patients and to identify potential clinical factors associated with these infections. The study question was framed using the PEO structure, as follows:

- Population (P): patients with lung cancer, including small cell lung cancer (SCLC) and non-small cell lung cancer (NSCLC) subtypes;
- Exposure (E): pulmonary colonization by pathogenic fungal species;
- Outcome (O): prevalence and clinical co-occurrence of FPIs-primarily CPA and PJP-in lung cancer patients.

The decision to focus on CPA and PJP as outcomes of interest was based on a pilot systematic search, which showed that these infections were the only ones consistently addressed as standalone topics in full-length observational studies. Other FPIs were predominantly described in isolated case reports.

### Systematic Search Strategy

A systematic literature search was conducted across five major databases: EBSCOhost, Embase, PubMed/MEDLINE, Scopus, and Web of Science. The predefined search strategy combined four primary keyword domains and their synonyms: fungal pulmonary infection, respiratory tract, lung cancer, and clinical outcome. Detailed PubMed/MEDLINE queries are provided in Supplementary Table S1, with corresponding strategies for EBSCOhost, Embase, Scopus, and Web of Science in Supplementary Tables S2-S5. 

The review followed a registered protocol (PROSPERO ID: CRD42024551104); however, the present analysis specifically focused on FPIs as a targeted subset of the broader review. The search and reporting processes adhered to the Preferred Reporting Items for Systematic Reviews and Meta-Analyses (PRISMA) 2020 guidelines.^12^

### Eligibility Criteria

Studies were eligible for inclusion if they:

- Investigated the association between clinical factors and fungal pulmonary infections (FPIs) in lung cancer patients;
- Were observational in design, including prospective or retrospective cohorts, case-control studies, or cross-sectional studies, provided they reported data separately for lung cancer patients with and without FPIs;
- Reported data on at least one of the following variables: demographics (age, sex, smoking history), comorbidities (e.g., cardiovascular disease, diabetes, underlying pulmonary disease), interventions (e.g., therapeutic regimens, surgical procedures), tumor histopathology, or cancer stage.

No restrictions were applied with regard to language or publication date. Records other than original research articles-including reviews, perspectives, editorials, and notes-as well as studies lacking the required data were excluded. Case reports were also excluded from the systematic review and meta-analysis in accordance with predefined criteria; however, a separate summary table of these case reports was compiled and included in the Discussion section to provide complementary insights into rare fungal infections and their clinical management.

### Study Screening and Data Extraction

Identified records were managed using Mendeley Desktop (version 1.19.8) (Mendeley, Elsevier, The Netherlands). Duplicate records were removed, and the studies were screened in two stages: (a) title and abstract screening to exclude irrelevant studies, and (b) full-text review to confirm eligibility. Data were extracted on study characteristics, effect measures, and relevant variables.

### Risk of Bias Assessment

The risk of bias in the included studies was assessed using the Risk Of Bias In Non-randomized Studies-of Interventions (ROBINS-I) tool,^13^ and the results were visualized with the RobVis package (https://mcguinlu.shinyapps.io/robvis/) to enhance transparency.^14^ ROBINS-I is the recommended instrument for evaluating bias in observational clinical studies of patient populations with defined conditions and treatment exposures,^13^ making it suitable for the present review. The tool covers seven domains: (1) confounding factors; (2) participant selection; (3) classification of interventions; (4) deviations from intended interventions; (5) missing data; (6) measurement of outcomes; and (7) selection of the reported result.^13^ Each domain was rated as having low, moderate, serious, or critical risk of bias.

### Statistical Analysis

A meta-analysis of proportions was conducted to estimate the prevalence of CPA and PJP in lung cancer patients using the meta package in RStudio (R version 4.2) under a random-effects model. Meta-analyses of dichotomous and continuous variables (clinical predictors) were performed separately for CPA and PJP using RevMan 5 (https://revman.cochrane.org/). A random-effects model was applied for variables exhibiting substantial heterogeneity (I² > 60%), while a fixed-effects model was used for those with moderate or low heterogeneity (I² ≤ 60%). All analyses used the restricted maximum-likelihood (REML) estimator, with statistical significance set at p<0.05. Heterogeneity was assessed with the I² statistic and its corresponding p-value. Results are presented in data tables and weighted forest plots. CPA and PJP were treated as distinct subgroups in all analyses. Only clinically relevant and statistically significant findings are shown as forest plots in the main text; complete sets of plots for all variables are available in the Supplementary Material.

## RESULTS

### Systematic Search

Supplementary Figure S1 presents the PRISMA 2020 flowchart of the systematic search. A total of 2,823 records were identified across EBSCOhost, Embase, PubMed/MEDLINE, Scopus, and Web of Science. After the removal of 91 duplicates, 2,732 records remained for title and abstract screening, of which 2,693 were excluded for not meeting the inclusion criteria. The full texts of the remaining 39 publications were reviewed, yielding 36 potentially eligible studies. Of these, 29 were excluded due to the absence of patient grouping based on the presence or absence of FPIs. Ultimately, 7 studies were included for assessment.

Table 1 summarizes the basic characteristics of the 7 included studies, which were published between 2009 and 2024. Most were observational studies conducted in Asian populations, with additional data from the UK, involving a total of 15,901 lung cancer patients, of whom 312 had concurrent FPIs: 177 CPA cases (4 studies) and 135 PJP cases (3 studies). Diagnoses were based on clinical signs and symptoms, supported by computed tomography (CT) imaging and sputum or bronchoalveolar lavage fluid (BALF) testing, including microbial culture and/or PCR. All CPA studies identified *Aspergillus fumigatus* as the causative strain, except Tamura et al.,^15^ who also isolated *Aspergillus niger*. 

**Table 1:** Baseline characteristics of the studies included in the systematic review. “Cases” refer to lung cancer patients diagnosed with either chronic pulmonary aspergillosis (CPA) or *Pneumocystis jirovecii* pneumonia (PJP); “controls” refer to lung cancer patients without these infections.

Study	Year	Country	Study Type	Pulmonary Infection Classification			Sample Size			Age	Ref.
				Type	Pathogen	Diagnostic Test(s)	Case	Control	Total		
Whittaker et al.	2024	UK	RSO	CPA	*A. fumigatus*	BALF/Sputum culture *Aspergillus* IgG	11	4,414	4,425	66.7 ± 10.8	(16)
Kim et al.	2022	South Korea	RSO	CPA	*A. fumigatus*	*A. fumigatus* IgG BALF/Sputum culture	93	6,684	6,777	62.7 ± 9.6	(17)
Zaini et al.	2022	Indonesia	CS	PJP	*P. jirovecii*	BALF/Sputum PCR	10	46	56	≥ 18	(18)
Shin et al.	2020	South Korea	RSO	CPA	*A. fumigatus*	*A. fumigatus* IgG BALF/Sputum culture	56	3,367	3,423	62.7 ± 9.4	(19)
Lee et al.	2019	South Korea	RSO	PJP	*P. jirovecii*	BALF/Sputum PCR/DFA	112	336	448	69 (42 - 88)	(20)
Tamura et al.	2015	Japan	RSO	CPA	*A. fumigatus* *A. niger*	Serum precipitin test BALF/Sputum culture	17	458	475	67 (24 - 86)	(15)
Nishigaki et al.	2009	Japan	RSO	PJP	*P. jirovecii*	BALF/Sputum PCR	13	284	297	72 (34 - 99)	(21)
Overall	−	−	−	−	−	−	312	15,589	15,901	−	−

BALF, bronchoalveolar lavage fluid; CPA, chronic pulmonary aspergillosis; CS, cross-sectional; RSO, retrospective observational; CT, computed tomography; DFA, direct fluorescent antibody; IgG, immunoglobulin G; IPA, invasive pulmonary aspergillosis; ODI, optical density index; PCR, polymerase chain reaction; PJP, *Pneumocystis jirovecii* pneumonia.

### Prevalence of CPA and PJP in Lung Cancer Patients

**Figure 1:**
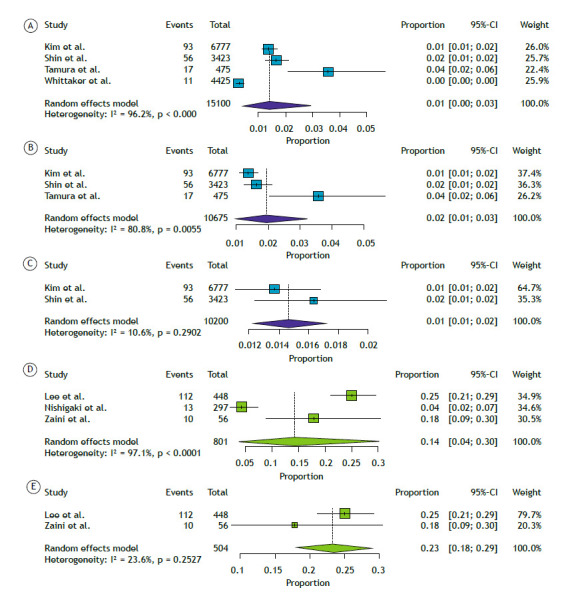
Meta-analysis of proportions for CPA and PJP. Panels A-C present results from 4, 3, and 2 studies on CPA, respectively, while panels D-E present results from 3 and 2 studies on PJP, respectively.

Meta-analyses of proportions for the CPA and PJP subgroups are presented in Figure 1. The initial analyses, which included all 4 studies on CPA and all 3 on PJP, showed substantial heterogeneity. In order to address this, studies contributing disproportionately to heterogeneity were sequentially excluded until acceptable heterogeneity levels were achieved. The final pooled prevalence estimates were 1% for CPA (95%CI: [0.01-0.02]; I² = 10.6%) based on a total sample size of 10,200 patients, and 23% for PJP (95%CI: [0.18-0.29]; I² = 23.6%) based on 504 patients.

### Prevalence of Demographics and Clinical Factors in CPA and PJP Subgroups of Lung Cancer Patients

Table 2 presents the results of the meta-analysis of proportions for demographic and clinical factors among lung cancer patients with CPA and PJP. Corresponding forest plots are shown in Supplementary Figures S2-S25.

**Table 2:** Meta-analysis of proportions. Prevalence of demographic and clinical variables among lung cancer patients with chronic pulmonary aspergillosis (CPA) or *Pneumocystis jirovecii* pneumonia (PJP), presented as distinct subgroups.

Subgroup	Variable		Proportion Rate		Statistic		Heterogeneity	
			Mean	95% CI	Z	P	I^2^ (%)	P
CPA	Sex	Female	0.17	[0.03, 0.31]	2.30	0.021	85.34	0.019
		Male	0.83	[0.69, 0.97]	11.20	< 0.001	85.34	0.019
	Comorbidity	DM	0.14	[0.08, 0.20]	4.49	< 0.001	24.97	0.296
		COPD	0.41	[0.31, 0.51]	7.95	< 0.001	38.74	0.148
		ILD	0.04	[0.01, 0.08]	2.81	0.005	0.29	0.168
		PTb	0.16	[0.10, 0.21]	5.53	< 0.001	0.00	0.874
		CHD	0.06	[0.02, 0.09]	3.11	0.002	0.10	0.079
		CVD	0.03	[0.00, 0.05]	2.20	0.028	0.00	0.641
	Smoking		0.87	[0.82, 0.97]	34.40	< 0.001	0.00	0.529
	Tumor Location	Left Lung	0.46	[0.38, 0.53]	12.20	< 0.001	0.00	0.805
		Right Lung	0.54	[0.47, 0.62]	14.50	< 0.001	0.00	0.805
		ADC	0.48	[0.41, 0.56]	12.40	< 0.001	0.00	0.816
		SCC	0.41	[0.33, 0.48]	10.80	< 0.001	0.00	0.555
	Tumor Stage	I-II	0.63	[0.48, 0.78]	8.20	< 0.001	73.35	0.020
		III-IV	0.37	[0.22, 0.52]	4.81	< 0.001	73.35	0.020
	Surgical Technique	Lobectomy	0.67	[0.30, 1.00]	3.52	< 0.001	97.89	< 0.001
		Bilobectomy	0.10	[0.05, 0.14]	4.26	< 0.001	0.00	0.641
		Pneumonectomy	0.01	[0.00, 0.03]	1.48	0.139	0.00	0.833
PJP	Sex	Female	0.20	[0.01, 0.40]	2.09	0.037	79.29	0.052
		Male	0.80	[0.60, 0.99]	8.11	< 0.001	79.29	0.052
	Tumor Histopathology	SCLC	0.11	[0.05, 0.16]	4.02	< 0.001	0.00	0.484
		NSCLC	0.89	[0.80, 0.97]	20.40	< 0.001	45.89	0.167
	Tumor Stage	IA-IIIA	0.26	[0.19, 0.34]	6.98	< 0.001	0.00	0.586
		IIIB-IV	0.74	[0.66, 0.81]	19.50	< 0.001	0.00	0.586

Legend: CPA, chronic pulmonary aspergillosis; PJP, *Pneumocystis jirovecii* pneumonia; DM, diabetes mellitus; COPD, chronic obstructive pulmonary disease; ILD, interstitial lung disease; PTb, pulmonary tuberculosis; CHD, chronic heart disease; CVD, cerebrovascular disease; ADC, adenocarcinoma; SCC, squamous cell carcinoma; SCLC, small cell lung cancer; NSCLC, non-small cell lung cancer.

Among the CPA patients, males predominated, with a pooled mean prevalence of 83% (95%CI: [0.69-0.97]). Comorbidities were generally less common in this subpopulation, except for chronic obstructive pulmonary disease (COPD), which had a pooled mean prevalence of 41% (95%CI: [0.31-0.51]). A positive smoking history was highly prevalent (87%). Tumor localization and histopathology were largely comparable across patients, while early-stage disease (stages I-II) was more frequent than advanced-stage disease (stages III-IV) (63% vs. 37%, respectively). Surgical management in this subgroup was predominantly lobectomy, with a pooled mean prevalence of 67% (95%CI: [0.30-1.00]). These findings are summarized in Table 2.

Studies involving lung cancer patients with PJP generally reported fewer clinical variables, resulting in a narrower breadth of findings. Similar to CPA, males predominated among PJP patients, with a pooled mean prevalence of 80% (95%CI: [0.60-0.99]; Table 2). Regarding tumor histopathology, NSCLC, including adenocarcinoma (ADC) and squamous cell carcinoma (SCC), was far more frequent than SCLC (89% vs. 11%, respectively). In contrast to CPA, advanced disease was found to be more prevalent in this subgroup, with stage IIIB-IV tumors accounting for 74% of cases.

### Meta-Analysis of Clinical Predictors of CPA and PJP in Lung Cancer Patients

**Figure 2:**
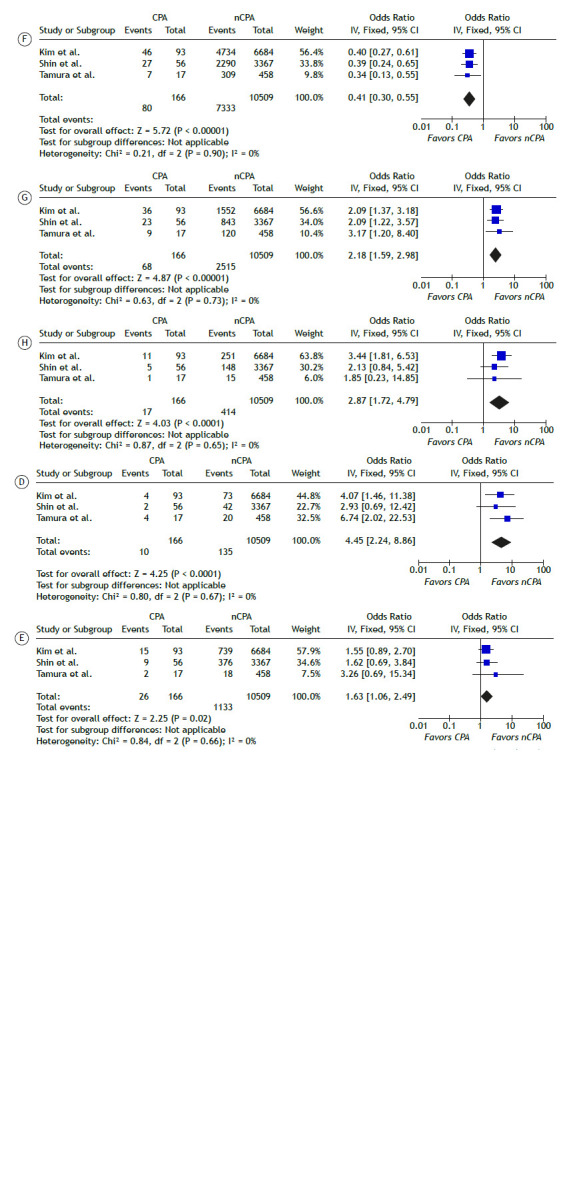
Meta-analysis of demographic and clinical predictors of CPA. Comparative forest plots of clinical and demographic factors showing statistically significant differences between CPA and non-CPA (nCPA) lung cancer patients, with low heterogeneity. Factors include male sex (A), current smoking (B), body mass index (C), interstitial lung disease (D), pulmonary tuberculosis (E), lung adenocarcinoma (F), lung squamous cell carcinoma (G), and bilobectomy (H).

A meta-analysis of dichotomous and continuous outcomes comparing CPA vs. non-CPA and PJP vs. non-PJP lung cancer patients is presented in Table 3, with statistically significant findings visualized as forest plots in Figure 2.

**Table 3:** Meta-analysis of demographic and clinical variables in lung cancer patients with fungal pulmonary infections. Comparisons were made between lung cancer patients with chronic pulmonary aspergillosis (CPA) vs. non-CPA (nCPA), and lung cancer patients with *Pneumocystis jirovecii* pneumonia (PJP) vs. non-PJP (nPJP). Odds ratios (OR) and standardized mean differences (SMD) represent dichotomous and continuous outcomes, respectively.

Subgroup	Variable		OR/SMD		Statistic		Heterogeneity	
			Mean	95% CI	Z	P	I^2^ (%)	P
CPA	Age		-0.02	[-0.17, 0.12]	-0.328	0.743	0.00	0.966
	Sex	Female	0.32	[0.14, 0.72]	-2.75	0.006	62.72	0.061
		Male	3.11	[1.38, 6.98]	2.75	0.006	62.72	0.061
	BMI (kg/m^2^)		-0.52	[-0.67, -0.36]	-6.48	< 0.001	0.00	0.521
	Smoking	Never	0.25	[0.17, 0.39]	6.29	< 0.001	0.00	0.530
		Ex	1.73	[1.25, 2.39]	3.28	0.001	0.00	0.810
		Current	1.88	[1.35, 2.61]	3.75	< 0.001	0.00	0.360
	Comorbidities	DM	1.03	[0.69, 1.54]	0.14	0.888	0.00	0.847
		COPD	2.22	[1.07, 4.56]	2.16	0.031	75.32	0.012
		ILD	4.45	[2.23, 8.86]	4.25	< 0.001	0.00	0.670
		PTb	1.63	[1.06, 2.49]	2.25	0.025	0.00	0.658
		CHD	1.31	[0.47, 3.68]	0.52	0.602	63.09	0.071
		CVD	0.65	[0.28, 1.53]	-0.98	0.327	0.00	0.875
	Tumor Location	Left Lung	1.18	[0.88, 1.59]	1.09	0.274	0.00	0.846
		Right Lung	0.85	[0.63, 1.14]	-1.09	0.274	0.00	0.846
	Tumor Histopathology	ADC	0.41	[0.30, 0.55]	-5.72	< 0.001	0.00	0.899
		SCC	2.18	[1.58, 2.98]	4.87	< 0.001	0.00	0.730
	Tumor Stage	I-II	0.47	[0.14, 1.53]	-0.12	0.210	90.60	< 0.001
		III-IV	2.13	[0.65, 6.98]	1.25	0.210	90.60	< 0.001
	Surgical Procedure	Lobectomy	1.29	[0.87, 1.92]	1.26	0.209	0.00	0.872
		Bilobectomy	2.87	[1.72, 4.79]	4.03	< 0.001	0.00	0.649
		Pneumonectomy	0.43	[0.12, 1.52]	-1.31	0.191	0.00	0.812
PJP	Sex	Female	1.00	[0.61, 1.68]	0.035	0.972	0.00	0.331
		Male	0.99	[0.59, 1.65]	-0.035	0.972	0.00	0.331
	Tumor Histopathology	SCLC	0.83	[0.46, 1.51]	-0.60	0.550	0.00	0.955
		NSCLC	0.90	[0.52, 1.56]	-0.36	0.719	0.00	0.829
	Tumor Stage	IA-IIIA	0.95	[0.62, 1.47]	-0.21	0.835	0.00	0.700
		IIIB-IV	1.06	[0.69, 1.64]	0.26	0.792	0.00	0.716

Legend: CPA, chronic pulmonary aspergillosis; PJP, *Pneumocystis jirovecii* pneumonia; BMI, body mass index; DM, diabetes mellitus; COPD, chronic obstructive pulmonary disease; ILD, interstitial lung disease; PTb, pulmonary tuberculosis; CHD, chronic heart disease; CVD, cerebrovascular disease; ADC, adenocarcinoma; SCC, squamous cell carcinoma; SCLC, small cell lung cancer; NSCLC, non-small cell lung cancer; OR, odds ratio; SMD, standardized mean difference.

Compared with non-CPA patients, CPA patients had significantly higher odds of male sex (OR: 3.11, 95%CI: [1.38-6.98]), smoking (OR: 3.92, 95%CI: [2.56-6.00]), COPD (OR: 2.22, 95%CI: [1.07-4.56]), interstitial lung disease (OR: 4.45, 95%CI: [2.23-8.86]), pulmonary tuberculosis (OR: 1.63, 95%CI: [1.06-2.49]), and squamous cell carcinoma (OR: 2.20, 95%CI: [1.60-2.98]). Conversely, adenocarcinoma was associated with significantly lower odds in CPA patients (OR: 0.41, 95%CI: [0.30-0.55]). 

For body mass index (BMI), a continuous variable, the meta-analysis yielded a statistically significant standardized mean difference (SMD) of -0.52 (95%CI: [-0.67 to -0.36]), indicating lower mean BMI in CPA patients compared to non-CPA patients. Heterogeneity was generally low for most statistically significant results, except for male sex and COPD, which showed moderately high levels of heterogeneity (I² > 60%).

Table 4 summarizes the results of the meta-analysis of demographic and clinical variables in PJP vs. non-PJP patients. Corresponding forest plots are provided in Supplementary Figures S26-S31. Unlike the CPA/non-CPA analysis, no clinical variables differed significantly between PJP and non-PJP patients. Odds ratios for sex (male/female), tumor histopathology (SCLC/NSCLC), and tumor stage (early/late) were broadly comparable, underscoring the need for further clinical investigations in this particular subgroup.

**Table 4:** Cases of fungal pulmonary infections (FPIs) in patients with lung cancer reported within the past two decades.

Study	Year	Country	Patient		Lung Cancer		Cancer Treatment History	Fungal Pulmonary Infection			Recommended Treatment	Ref.
			Age	Sex	Type	Stage		Type	Pathogen	Diagnostic Test		
Bai et al.	2024	China	54	Female	ADC	IA	−	PC	*Cryptococcus* spp.	PAS/GMS/MC stain	Fluconazole (PO, 200 mg, OD, 3 months)	(42)
Ng at al.	2024	Malaysia	73	Male	ADC	−	Chemotherapy	Aspergillosis	*A. fumigatus*	PAS/GMS stain	AMB (IV, 2 weeks)	(43)
											Voriconazole (PO, 6 weeks)	
Setiwalidi et al.	2024	China	56	Male	ADC	IIIA	Pralestinib Selpercatinib	Polymicrobial	*A. fumigatus* *C. albicans* *P. jirovecii*	1,3-β-D-Glucan + Galactomannan antigen test	Voriconazole (PO, 12 weeks)	(44)
Guziejko et al.	2022	Poland	54	Female	ADC	IIIA	Etoposide Radiotherapy	CPA	*A. fumigatus* *A. flavus*	ODID	Itraconazole (PO, 200 mg, BID, 3 months)	(45)
											Voriconazole (PO, 200 mg, BID)	
Hiba et al.	2022	Morocco	50	Female	ADC	−	Carboplatin Paclitaxel Pembrolizumab Corticosteroids	PJP	*P. jirovecii*	BALF examination*	TMP/SMX	(46)
Kim et al.	2022	South Korea	64	Male	ADC	IA	Lobectomy	PJP	*P. jirovecii*	BALF PCR	TMP/SMX (IV, 320/160 mg, TID)	(47)
Doello et al.	2020	Spain	59	Male	NSCLC	IIIB	Cisplatin Vinorelbine Radiotherapy	PJP	*P. jirovecii*	BALF examination*	TMP/SMX (IV, 160/800 mg, TID)	(48)
											Voriconazole (PO, 300 mg daily)	
Yao et al.	2020	China	72	Male	SCC	−	−	PC	*Cryptococcus* spp.	Puncture histopathology	Fluconazole (PO, 400 mg daily, 6 months)	(49)
Uehara et al.	2018	Japan	70	Male	SCC	−	Lobectomy	CPA	*A. fumigatus*	1,3-β-D-Glucan + Galactomannan antigen test	Micafungin	(50)
											Voriconazole (PO, 200 mg, BID)	
Boyd et al.	2013	US	48	Male	ADC	IA	−	IPA	*Aspergillus* spp.	Histopathology*	Voriconazole	(51)
Santos et al.	2013	Brazil	76	Male	ADC	−	Cisplatin Dexamethasone Pemetrexed disodium	IPA	*A. fumigatus*	BALF PCR	Voriconazole (IV, 4 mg/kg, BID, 3 weeks)	(52)
											Voriconazole (PO, 200 mg, BID)	
Uchida et al.	2012	Japan	74	Male	SCLC	IIIB	−	Mucormycosis	*C. bertholletiae*	BALF culture	Liposomal AMB (2.5 mg/kg, 1 month)	(53)
Neuville et al.	2011	France	49	Male	ADC	−	Gemcitabine Carboplatin Bevacizumab Pemetrexed disodium	PJP	*P. jirovecii*	BALF PCR	TMP/SMX (IV, 400/80 mg, OD, 3 weeks)	(54)
Velcheti et al.	2007	US	73	Male	NSCLC	−	Gemcitabine Pemetrexed disodium Radiotherapy	PJP	*P. jirovecii*	BALF-DFA	TMP/SMX (IV, 400 mg, QID)	(55)
Itano et al.	2005	Japan	77	Male	ADC	IA	−	IPA	*Aspergillus* spp.	Histopathology*	Itraconazole (PO, 100 mg, OD, 2 weeks)	(56)

Legend: *Exact diagnostic test was not specified. ADC, adenocarcinoma; NSCLC: non-small cell lung cancer; SCC: squamous cell carcinoma; SCLC: small cell lung cancer; PC: pulmonary cryptococcosis; CPA: chronic pulmonary aspergillosis; PJP, *Pneumocystis jirovecii* pneumonia; IPA: invasive pulmonary aspergillosis; SIPA: subacute invasive pulmonary aspergillosis; PAS: periodic acid Schiff; GMS: Gomori methenamine silver; MC: mucicarmine; ODID: Ouchterlony double immunodiffusion; BALF: bronchoalveolar lavage fluid; PCR: polymerase chain reaction; DFA: direct fluorescent antibody; PO: per os (oral); OD: once daily; AMB: amphotericin B; BID: twice daily; TMP/SMX: trimethoprim/sulfamethoxazole; IV: intravenous; TID: three times daily; QID: four times daily.

### Treatment-Associated Risk of CPA in Lung Cancer Patients

**Figure 3:**
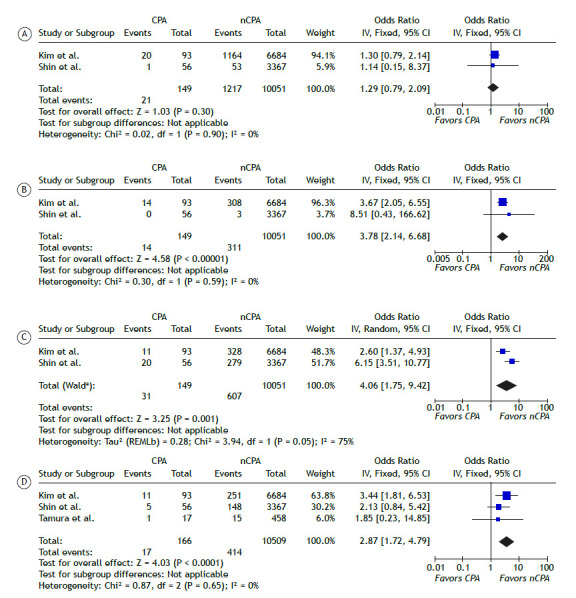
Meta-analysis of therapeutic predictors of CPA. Forest plots comparing chemotherapy (A), radiotherapy (B), concurrent chemoradiotherapy (CCRT) (C), and bilobectomy (D) between CPA and non-CPA lung cancer patients.

As shown in Figure 3, chemotherapy was not significantly associated with CPA in patients with lung cancer (OR: 1.29; 95%CI: [0.79-2.09]). In contrast, chest radiotherapy showed a significant association with CPA (p<0.001), with a pooled OR of 3.78 (95%CI: [2.14-6.68]) and negligible heterogeneity (I² = 0%). Concurrent chemoradiotherapy (CCRT) also showed a significant association (p=0.001), with a pooled OR of 4.06 (95%CI: [1.75-9.42]), though with substantial heterogeneity (I² = 75%). 

Bilobectomy-defined as the surgical removal of two tumor-bearing lobes-emerged as a significant predictor of CPA, with a pooled OR of 2.87 (95%CI: [1.72-4.79]) and low heterogeneity (I² = 0%). Other surgical procedures, including lobectomy (single-lobe resection) and pneumonectomy, were not significantly associated with CPA (Table 3). 

Collectively, these findings suggest that radiotherapy (alone or combined with chemotherapy) and bilobectomy may represent important risk factors for CPA development in lung cancer patients.

### Treatment-Associated Risk of PJP in Lung Cancer Patients

**Figure 4:**
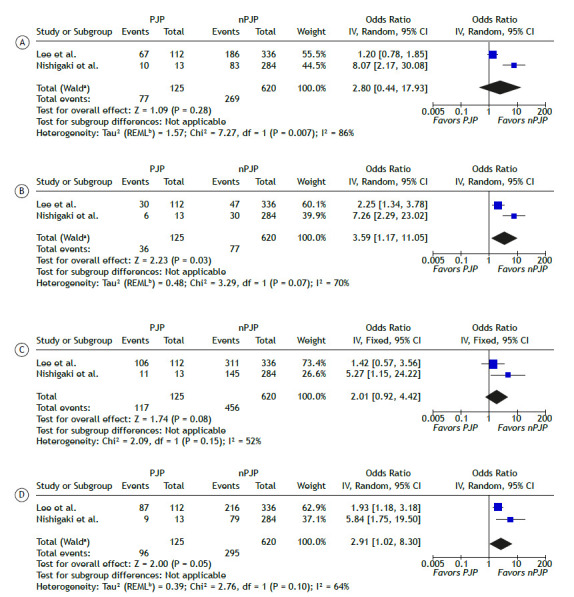
Meta-analysis of therapeutic predictors of PJP. Forest plots comparing corticosteroid therapy (A), high-dose corticosteroid therapy (B), chemotherapy (C), and radiotherapy (D) between PJP and non-PJP lung cancer patients.

Overall, corticosteroid therapy was not significantly associated with PJP in lung cancer patients (OR: 2.80; 95%CI: [0.44-17.39]; Figure 4). However, high-dose corticosteroid use-defined as a daily dose of ≥20 mg-was significantly associated with increased odds of PJP (p=0.03), with a pooled OR of 3.59 (95%CI: [1.17-11.05]) and moderately high heterogeneity (I² = 70%).

Consistent with the CPA findings, chemotherapy was not significantly associated with PJP (p=0.08). Radiotherapy, on the other hand, showed a borderline significant association with PJP (p=0.05), with a pooled OR of 2.91 (95%CI: [1.02-8.30]) and moderate heterogeneity (I² = 64%).

### Risk of Bias Assessment

The risk of bias in the included studies was assessed using the ROBINS-I tool. As shown in Supplementary Figure S32, most studies exhibited low risk of bias across the majority of domains. The main concerns were related to confounding factors (D1) and participant selection (D2), where several studies were judged to have moderate risk. Key confounders included age, sex, and smoking history between lung cancer patients with and without FPIs. 

Participant selection was deemed to confer serious risk of bias in the study by Lee et al.,^20^ which recruited patients with confirmed PJP and compared them with a cohort of lung cancer patients without PJP. Furthermore, the study by Zaini et al.^18^ had missing information in certain domains, particularly regarding deviations from intended interventions and classification of interventions, mostly involving surgical procedures and therapeutic regimens in PJP and non-PJP groups. Overall, most studies were considered to have a moderate risk of bias.

## DISCUSSION

This systematic review and meta-analysis evaluated the prevalence of fungal pulmonary infections (CPA and PJP) in 15,901 lung cancer patients across seven studies, identifying 312 cases. Key clinical associations were also explored to support risk stratification and management.

### Prevalence and Clinical Context

After excluding heterogeneous studies, the pooled prevalence rates were 1% for CPA and 23% for PJP, indicating a higher frequency of PJP. The clinical association between PJP and immunocompromised states^22^ contrasts with CPA’s association with structural lung damage,^23^ which may partly explain the lower prevalence of CPA, despite the widespread use of chemotherapy in lung cancer patients.^24^ A Chinese study reported fungal infections in 28.7% of patients based on sputum cultures, with a higher proportion having a history of radiotherapy (31.3% vs. 18.5%).^25^ Japanese data showed 31% fungal PCR positivity in lung cancer patients, attributed largely to corticosteroid use.^26^ Notably, PJP can arise without prior colonization, as observed in four cases without preceding fungal detection.^27^ Emerging antifungal resistance, such as voriconazole resistance in 42.4% of *Aspergillus* isolates in Indonesia,^28^ underscores the clinical relevance of FPIs despite the modest 1% prevalence of CPA reported in this meta-analysis.

### Clinical Predictors of CPA

Male sex, COPD, interstitial lung disease (ILD), and SCC histology were positively associated with CPA, while lower BMI showed an inverse correlation. A 2024 cohort study linked lung cancer to CPA, with a hazard ratio of 8.51.^29^ Similarly, a nationwide Japanese observational study identified lung cancer, COPD, and ILD as major risk factors for CPA,^30^ consistent with our findings. A large-scale French analysis of 17,290 CPA cases over a 10-year period further corroborated these associations.^31^ In addition, a Spanish study reported a 9.5-fold increase in mortality among CPA patients with a history of lung cancer.^32^ SCC histology and BMI <18.5 kg/m² predicted poorer survival in CPA patients,^6^ aligning with our meta-analysis, showing higher odds of SCC and lower BMI in CPA cases. The clinical relevance of low BMI is further supported by an 11-year retrospective study from Brazil involving 91 CPA patients, which found a predominance of underweight individuals, reinforcing the association between low BMI and CPA susceptibility.^33^ Notably, a large-scale cohort study of 7,021 patients with advanced NSCLC showed improved overall survival in obese patients receiving chemotherapy or immunotherapy compared with those of normal BMI,^34^ further highlighting the prognostic implications of body weight in lung cancer populations.

### Treatment-Related Associations

Radiotherapy and CCRT were significantly associated with CPA. Chest radiotherapy, particularly when combined with chemotherapy, increases the risk of fungal infection, with the highest incidence occurring within three months of treatment initiation.^35^ CCRT is especially linked to aspergillosis, with susceptibility peaking during the first three months of treatment. In a cohort of 4,450 patients, fungal infections were reported in 15.9% post-radiotherapy, with markedly higher rates in CCRT patients (60.5% vs. 39.5%).^36^ These findings are consistent with our meta-analysis, which showed higher odds of CPA in lung cancer patients with prior CCRT exposure compared to those receiving radiotherapy alone (4.06 vs. 3.78), suggesting that CCRT confers an added risk. A two-year follow-up of 1,872 lung cancer patients undergoing radiotherapy identified CPA in 24 out of 54 cases (44.4%) of chronic pulmonary infections, establishing CPA as the predominant type in this setting.^37^ Consistently, a two-decade retrospective survey in Japan of 187 NSCLC patients receiving postoperative CCRT reported CPA in 6 cases (3.2%).^10^ This prevalence is approximately three times higher than the 1% weighted mean observed in our meta-analysis, underscoring the contributory role of CCRT in CPA development. The increased odds of CPA following radiotherapy may be partly explained by radiation-induced pneumonitis,^38^ compounded by immunosuppression associated with CCRT, further facilitating fungal colonization and the occurrence of CPA.^10^

### PJP and Corticosteroid Use

High-dose corticosteroids (≥20 mg/day) were strongly associated with PJP, whereas lower dosages showed non-significant trends. The association between corticosteroid therapy and PJP is well-established in the literature.^39^ Our analysis revealed a pooled mean OR of 2.80 (95%CI: [0.44-17.93]; Figure 4) for PJP in patients with a history of corticosteroid treatment, irrespective of dosage; however, this association did not reach statistical significance. In contrast, daily doses ≥20 mg were significantly associated with PJP. This finding is clinically relevant, as demonstrated by the 2024 PCP-MULTI group study, which reported that among 66 solid tumor patients with PJP, 44 (66.7%) were receiving corticosteroids at daily doses ≥40 mg at the time of infection. High-dose corticosteroid use was linked to significantly lower survival.^40^ Collectively, these data support a dose-dependent risk of PJP, highlighting the importance of prophylaxis in patients receiving high-dose corticosteroid therapy.^41^

### Potential Implications of Observed Heterogeneity

Several instances of elevated heterogeneity were observed across our analyses, particularly in the meta-analyses of proportions. Initial pooled prevalence estimates for CPA and PJP showed substantial heterogeneity (I² > 90%), which was mitigated through subgroup analyses and stepwise exclusion of outlier studies. Residual high heterogeneity was mainly confined to descriptive variables, such as sex distribution and the proportion of lobectomy among CPA patients. While informative, these were not central to our clinical interpretations. 

The core of our findings lies in the meta-analyses of binomial outcomes for clinical predictors, where heterogeneity was generally low to moderate. Exceptions include COPD in CPA patients (I² = 75%) and tumor stage (I/II vs. III/IV), the latter retaining high heterogeneity (I² = 90%) despite *post-hoc* harmonization of stage definitions across a limited number of studies. Corticosteroid use in PJP patients also showed considerable heterogeneity (I² = 86%) in the overall analysis, though the association was not statistically significant (p=0.28). In contrast, high-dose corticosteroid use demonstrated a significant association with moderate heterogeneity (I² = 70%). These elevated values likely reflect the small number of available studies and the geographical homogeneity of included cohorts, the majority of which were conducted in Asia. Accordingly, we emphasize results with lower heterogeneity, which we consider more reliable and generalizable.

### Limitations and Generalizability to Diverse Populations

Our analysis is largely based on retrospective observational studies from Asia, reflecting the limited research on FPIs in lung cancer patients elsewhere. Isolated case reports from Europe, North America, Africa, and South America can be found in the literature. Table 4 provides a summary of pertinent case reports published since 2005, documenting the co-occurrence of pulmonary infections and lung cancer. These reports-spanning diverse histologies (ADC, NSCLC), stages (IA-IIIB), and treatments (platinum regimens, radiotherapy, immunotherapy, corticosteroids)-lack uniform denominators and standardized diagnostic criteria, undermining prevalence estimates outside Asia. 

In broader CPA cohorts, 1-year mortality ranges from 7% to 32%, and 5-year mortality from 38% to 52%, with pulmonary cavitation representing the key risk factor and CT imaging plus *Aspergillus* IgG serology remaining central to diagnosis and management.^57^ Similarly, non-HIV PJP occurs predominantly in immunocompromised hosts, with malignancy present in up to 46% of cases and systemic glucocorticoids in up to 76%, though without a clearly defined dose threshold.^58^ Our identification of high-dose corticosteroids (≥20 mg/day) as a predictor for PJP therefore offers novel, dose-specific insight. Nonetheless, in the absence of prospective cohorts from non-Asian regions applying consistent methodology, the external validity of our pooled prevalence and risk estimates remains constrained, underscoring the need for multinational studies with harmonized diagnostic and treatment protocols.

### Assessment of Publication Bias

A formal assessment of publication bias was carried out with caution, as the included outcomes involved <10 studies, which is commonly considered the minimum threshold for meaningful evaluation using funnel plots or Egger’s test.^59^ Funnel plots for CPA and PJP studies are presented in Supplementary Figures S31-S33. The funnel plot for all CPA studies showed asymmetry, with the study by Whittaker et al.^16^ identified as an outlier. Upon exclusion of this study, Egger’s regression yielded a Z score of 1.69 (p=0.090), indicating no significant asymmetry or publication bias. Egger’s regression for PJP studies produced a Z score of 0.664 (p=0.507), similarly suggesting no evidence of asymmetry or publication bias. Since the Whittaker et al.^16^ study was not included in most clinical predictor and treatment-related meta-analyses due to insufficient data (Figures 3-4), we can conclude that publication bias is unlikely to have substantially influenced our findings.

### Clinical Remarks and Practical Considerations

In routine practice, FPIs in lung cancer are investigated only when clinical or radiologic findings-such as new infiltrates, persistent fevers, or nodular lesions-raise suspicion, rather than through universal screening.^57^ ^,^ ^58^ For PJP, current guidelines recommend initiating prophylaxis once patients receive the equivalent of ≥20 mg prednisone daily for ≥4 weeks or,^60^ according to the 2022 European Alliance of Associations for Rheumatology (EULAR) update, ≥15 mg daily for ≥2 weeks.^57^ Trimethoprim-sulfamethoxazole (TMP/SMX) remains the first-line regimen, with dosing adjusted for renal function and desensitization protocols available for sulfa-allergic patients. Its efficacy is well supported, including in a recent risk-benefit analysis of primary prophylaxis against PJP involving 419 patients receiving TMP/SMX.^61^ Within lung cancer-associated case reports (Table 4), TMP/SMX consistently appears as the predominant treatment modality for PJP, reinforcing its central role. By contrast, CPA typically arises from structural lung damage-such as post-radiotherapy pneumonitis or bilobectomy-and routine antifungal prophylaxis is neither standard nor practical given concerns about toxicity, drug interactions, and cost.^57^ Instead, our findings suggest a risk-stratified surveillance approach: patients undergoing high-risk interventions, including bilateral lung resections or CCRT, may benefit from scheduled chest imaging or serum biomarkers (e.g., galactomannan, *Aspergillus* PCR; see Table 1) in the months following treatment to enable earlier CPA detection. Together, these observations support targeted PJP prophylaxis and individualized CPA monitoring strategies to optimize fungal infection management in lung cancer care.

## CONCLUSION

This systematic review and meta-analysis underscores the prevalence and clinical relevance of fungal pulmonary infections (FPIs) in lung cancer patients, focusing on chronic pulmonary aspergillosis (CPA) and *Pneumocystis jirovecii* pneumonia (PJP). PJP emerged as the predominant infection, with a pooled prevalence of 23% compared to 1% for CPA, reflecting their distinct pathogenic mechanisms-immunosuppression for PJP versus structural lung damage for CPA. Key clinical predictors of CPA included male sex, coexisting COPD or interstitial lung disease, squamous cell carcinoma (SCC) histology, low body mass index (BMI), and prior radiotherapy or chemoradiotherapy. The inverse association between BMI and CPA risk highlights the contribution of nutritional status. For PJP, corticosteroid use was the main risk factor, with daily doses ≥20 mg significantly increasing the risk of infection, reinforcing the need for dose-aware prophylactic strategies.

These findings support a risk-based approach to screening and prophylaxis, emphasizing multidisciplinary collaboration among oncologists, pulmonologists, and infectious disease specialists. Treatment decisions, particularly those involving corticosteroids and radiotherapy, should be carefully balanced against infection risk. Future research should aim to develop validated risk models and evaluate targeted prophylaxis, optimal therapeutic regimens, and long-term outcomes. FPIs represent a clinically significant complication in lung cancer, and tailoring management to their distinct risk profiles may improve prevention and patient care.
